# A phase II study of biweekly oxaliplatin plus S-1 combination chemotherapy as a first-line treatment for patients with metastatic or advanced gastric cancer in China

**DOI:** 10.1097/MD.0000000000015696

**Published:** 2019-05-17

**Authors:** Cheng Xiao, Jiong Qian, Yulong Zheng, Fang Song, Qiangfeng Wang, Haiping Jiang, Chenyu Mao, Nong Xu

**Affiliations:** a Department of Medical Oncology; b Department of Anesthesiology, the First Affiliated Hospital of Zhejiang University, Hangzhou, China.

**Keywords:** gastric cancer, biweekly, oxaliplatin, S-1, SOX, phase II

## Abstract

Oxaliplatin plus S-1 (SOX) was a first-line regimen for advanced gastric cancer. The continuous administration of S-1 for 3 weeks can result in unacceptable gastrointestinal and hematological toxicities. Therefore, an alternative regimen (administration of S-1 for 1-week followed by 1-week rest) is warrant for improved tolerability and noninferiority efficacy. We conducted a study to evaluate the efficacy and safety of biweekly SOX as the first-line chemotherapy in patients with metastatic or advanced gastric cancer in China.

Patients with metastatic or previously untreated advanced gastric cancer were enrolled. Oxaliplatin was administered intravenously at a dose of 85 mg/m^2^ on day 1, while S-1 was administered orally in doses of 80, 100, or 120 mg/day depending on different body surface areas of <1.25 m^2^, 1.25–1.5 m^2^, or >1.5 m^2^ respectively; the total dose of S-1 was administered twice daily on days 1–7 followed by a 7-day rest. This schedule was repeated every 2 weeks until disease progressed or intolerable toxicity occurred.

Forty-six patients (M/F = 33/13) received biweekly oxaliplatin and S-1 as first-line chemotherapy. A total of 257 treatment cycles were administered and the median number of cycles administered was 6. Thirty-six patients (78.3%) received second-line chemotherapy. The median progression free survival and median overall survival was 4.4 months (95% CI, 3.37–5.36 months) and 10.3 months (95% CI, 8.88–11.3 months), respectively. The 1-year and 2-year survival rate was 41% and 13%. The objective response rate was 30.43%, and the disease control rate was 76.08%. The observed adverse events of Grade 3/4 included were leukocytopenia (13.04%); anemia (13.04%); neutropenia (15.22%); neurological toxicity (2.17%); diarrhea (2.17%).

The biweekly SOX regimen as first-line treatment was active and well tolerated in Chinese patients with metastatic or advanced gastric cancer.

## 1 Introduction

Gastric cancer is one of the most frequent malignancy types of cancer. Radical surgery is the only curable treatment modality for patient with resectable disease. However, majority of the patients are diagnosed with advanced gastric cancer^[1]^ and 40% of patients will recur after curative resection.^[2]^ The median survival time for patients with advanced gastric cancer ranges from 8 to 16 months in previous studies.^[3–6]^ Basis on the results of the studies, systemic palliative chemotherapy combines fluorinated pyrimidines and platinum-based drugs improved their survival and quality of life for patients with metastatic or advanced gastric cancer.^[7,8]^

Oxaliplatin is a newer-generation platinum agent for the treatment of gastric cancer which is less adverse events besides gastrointestinal toxicities and peripheral sensory neuropathy.^[9]^ The REAL-2 study demonstrated that oxaliplatin was as effective as cisplatin.^[10]^ Another phase III study also reported that oxaliplatin plus 5-FU/leucovorin has a better progression-free survival (PFS) and overall survival time (OS) than cisplatin plus 5-FU/leucovorin.^[9]^ The National Comprehensive Cancer Network Guidelines suggested that oxaliplatin is a recommended regimen for patients with metastatic or advanced gastric cancer. S-1 is an orally administered prodrug of 5-fluorouracil, and the response rates of S-1 in the treatment of gastric cancer was 32%.^[11]^ The SPIRITS study indicated that cisplatin plus S-1 can served as the standard first-line therapy for advanced gastric cancer.^[3]^ In addition, another study showed that S-1 in combination with oxaliplatin demonstrate non-inferiority and well tolerated in Asian country.^[12]^ The FLAGS phase III study (S-1 plus cisplatin) carried out in Western countries also demonstrated that cisplatin plus S-1 was at least as effective as cisplatin plus 5-FU with a better safety profile.^[13]^ Wagner's study showed that oxaliplatin-containing regimens demonstrated a benefit in OS as compared to cisplatin-containing regimens, and there is a survival improvement of S-1 compared to 5-FU.^[8]^

There are several first-line chemotherapy regimens against metastatic gastric cancer. In China, metastatic or advanced gastric cancer is mainly treated with S-1combined with oxaliplatin, cisplatin, or paclitaxel. And SOX regimen was used more than SP for less adverse effect. Since the current clinical data on 3-weekly SOX regimen with an oxaliplatin dose of 100 or 130 mg/m^2^ and continuous administration of S-1 for 2 or more weeks resulted in unsatisfied toxicities such as neutropenia, thrombocytopenia, anemia, fatigue, and sensory neuropathy. The most optimization of schedule for S-1 and dose of oxaliplatin has yet to be set up. As a result, oncologists may shift their practice toward a more individualized treatment strategy with improved tolerability. We hypothesize that biweekly SOX may further reduce the toxicities compared to 3-weekly SOX. Given the scarcity of study on biweekly SOX as first-line chemotherapy in patients with metastatic or advanced gastric cancer, we performed this study of biweekly SOX as the first-line chemotherapy in patients with metastatic or advanced gastric cancer.

## 2 Methods/Design

### 2.1 Study setting

There were few data on biweekly SOX with an oxaliplatin dose of 85 mg/m^2^ to treat metastatic or advanced gastric cancer in China, so a clinical trial was conducted to examine the efficacy and safety of that therapy regimen.

### 2.2 Patients

All patients enrolled in the investigation had histologically confirmed Human Epidermal Growth Factor Receptor type 2 (HER2) negative metastatic or advanced gastric cancer and at least one measurable lesion according to Response Evaluation Criteria in Solid Tumors (RECIST) criteria version 1.1. Patients were eligible if they had completed adjuvant chemotherapy treatment 6 months ago. Other inclusion criteria were as follows: Eastern Cooperative Oncology Group (ECOG) performance status less than or equal to 2; predicted life expectancy at least 4 months; 18–75 years of age; adequate bone marrow: white blood cell count 3.0 ≥ × 10E^9^/L, absolute neutrophil count more than or equal to 1.5 × 10E^9^/L, platelet count more than or equal to 100 × 10E^9^/L, and hemoglobin more than or equal to 90 g/L; adequate hepatic functions: transaminase less than or equal to 3.0 times the upper normal limit (UNL) and serum bilirubin less than or equal to 1.5 × UNL; adequate renal functions: serum creatinine less than or equal to 135 μmol/L; adequate normal cardiac function.

The major exclusion criteria were as follows: active double cancer; pregnancy; a severe comorbidity; allergic to oxaliplatin or S-1; prior radiotherapy in parameter lesions.

Patients were required to provide written informed consent before participate in the study. This prospective clinical trial was approved by the ethical committee of the First Affiliated Hospital of Zhejiang University. The registry number of our study is ISRCTN85705844. The reference number of our study is: Approval Letter of Ethics Committee of the First Affiliated Hospital, College of Medicine, Zhejiang University 2016 No. 116.

### 2.3 Treatment protocol

Patients were treated with oxaliplatin (85 mg/m^2^) on day one plus S-1 (depending on patient's body surface area), twice daily on days 1 to 7 followed by a 7-day rest period chemotherapy. Treatment cycles were repeated every 2 weeks until disease progressed or intolerable toxicity occurred.

Toxicity of this study was graded according to The National Cancer Institute Common Toxicity Criteria version 3.0. In the event of grade 4 hematological toxicities or grade 3 gastrointestinal toxicities, the doses of oxaliplatin and S-1 were reduced 25% in the following cycle, and the doses could be reduced by two dose levels. Treatment was resumed until all toxicity recovery to grade 0 or 1. If the discontinuation of treatment was more than 2 weeks, the patient was excluded from the study. Second-line chemotherapy was permitted.

### 2.4 Follow-up evaluation and response assessment

We collected the data of physical examination and routine hematologic studies weekly. Abdomen and lung contrast enhanced computed tomography (CT) or magnetic resonance imaging (MRI) scans were conducted to evaluate the response after every four cycles of chemotherapy. If there was evidence of any clinical deterioration, we assess the treatment effects immediately. Responses were evaluated according to RECIST 1.1 criteria.^[14]^

### 2.5 Statistical methods

PFS and OS were calculated. PFS was calculated from the date of initiation of therapy to the date of first disease progression. OS was defined as the date from treatment initiation to the date of final follow-up or death. The primary objective of this study was to evaluate the overall response rate (ORR), and the secondary objective were to evaluate OS, PFS, disease control rate (DCR), toxicities, and safety. All data were analyzed using SPSS software (version 18.0, Chicago, IL). Kaplan–Meier method was used to analyze the median PFS and OS.

## 3 Results

### 3.1 Patient characteristics

Between Feb 2016 and Dec 2017, 46 patients received the treatment at the Department of Medicine Oncology, the First Affiliated Hospital of Zhejiang University, China. The male-to-female ratio was 33:13, and the median age was 59 years (range, 29–78 years). Forty-two patients were newly diagnosed as advanced gastric cancer and four patients evidenced gastric cancer relapse following radical operation. The most frequent metastatic sites were the liver (41.3%) and lymph node (39.13%). 86.96% of the patients had an ECOG performance status of 0 or 1. The baseline characteristics of the patients are listed in Table 1.

**Table 1:**
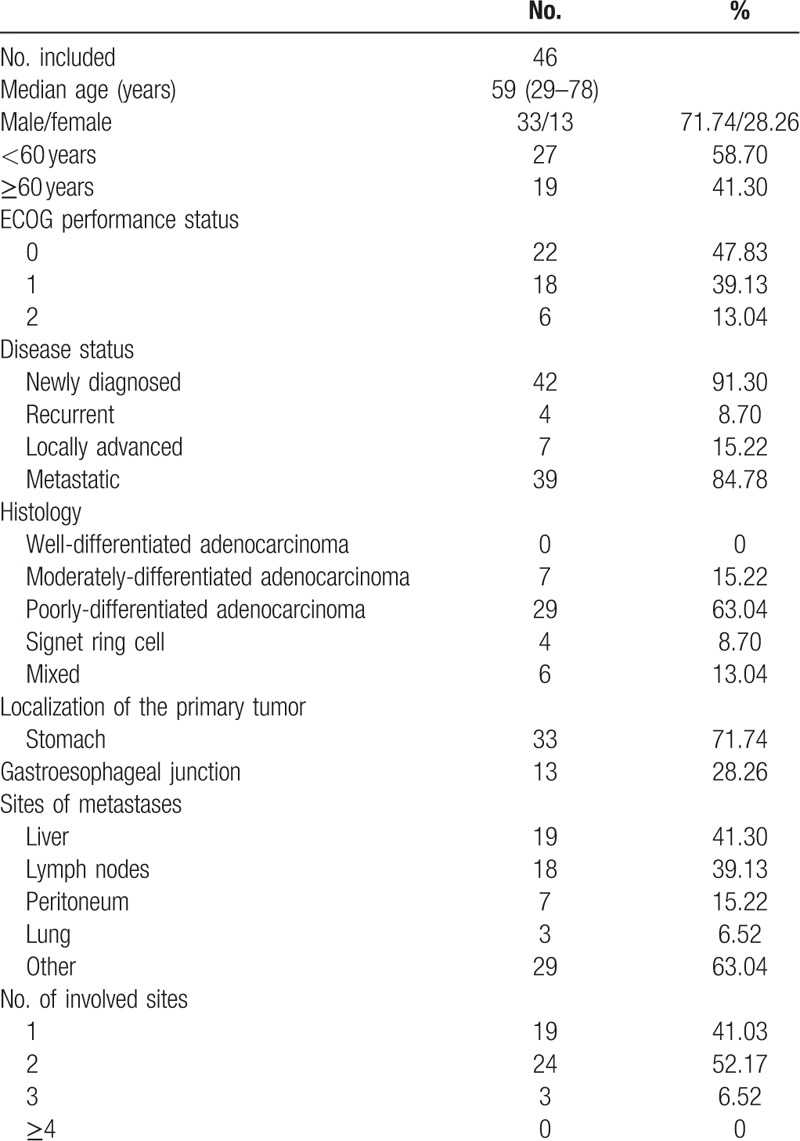
Baseline patient's characteristics (N = 46).

### 3.2 Response

There were 14 patients (30.43%) achieved a partial response (PR), 21 patients (45.65%) evidenced stable disease (SD), and 11patients (23.91%) progressed (PD) during the treatment. The assessed ORR was30.43% (14/46) and the DCR was 76.08% (35/46). None patient was lost to follow-up prior to evaluation. The results are listed in Table 2.

**Table 2:**
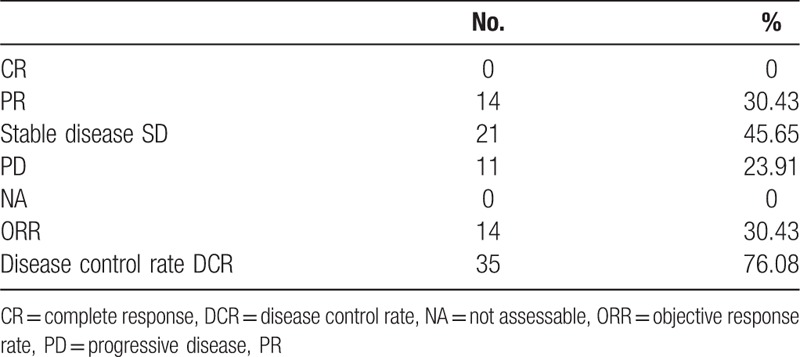
Best overall response (N = 46).

### 3.3 Survival

**Figure 1:**
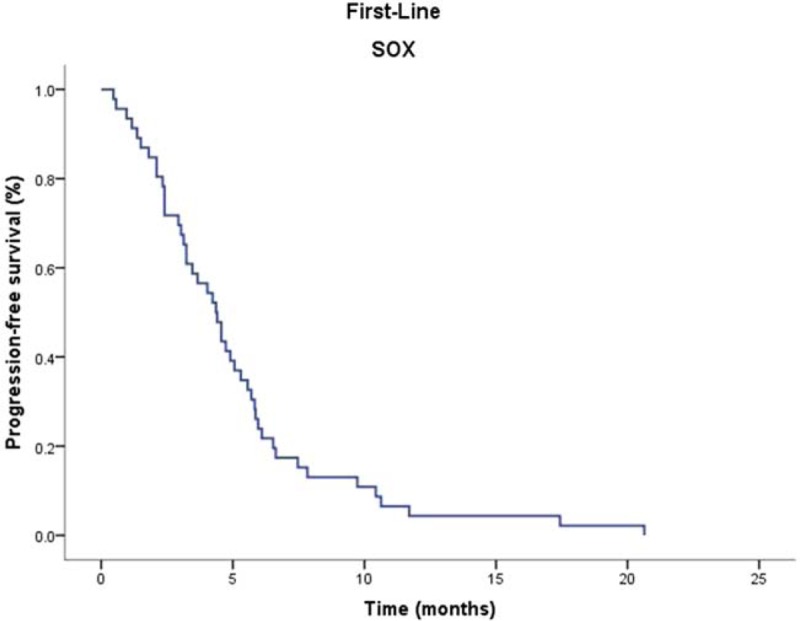
Kaplan–Meier curve for progression-free survival.

**Figure 2:**
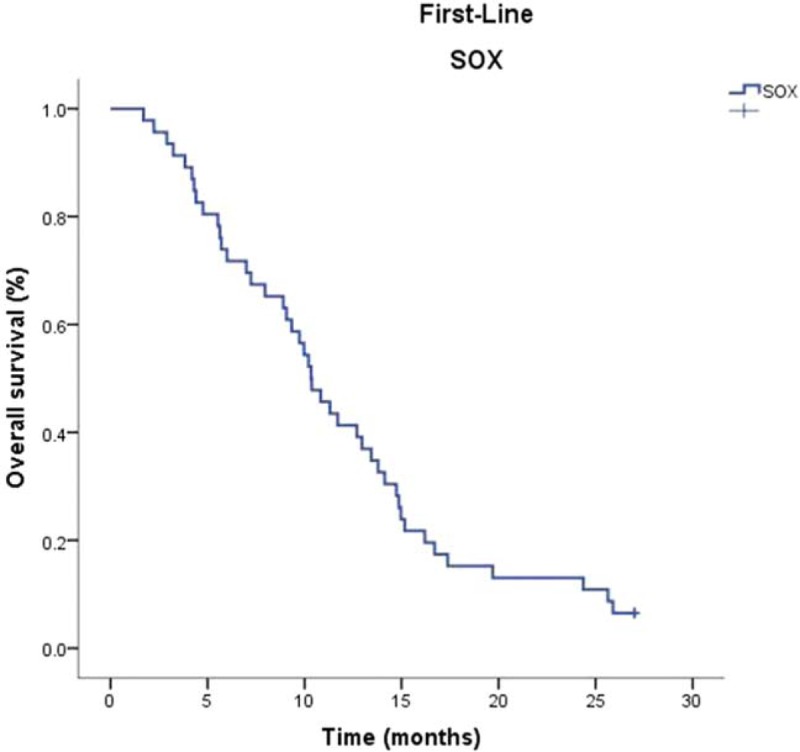
Kaplan–Meier curve for overall survival.

The median follow-up duration was 20.8 months. The PFS and OS were 4.4 months (95% CI, 3.37–5.36 months) and 10.3 months (95% CI, 8.88–11.3 months) respectively. PFS and OS were evaluated via Kaplan–Meier analysis, as shown in Figures 1 and 2. The one-year and two-year survival rates were 41% and 13%, respectively. The patients did not have any other disease or living habit highly relevant to the gastric cancer. There were 11 patients had progressive disease during the first-line chemotherapy. Forty-five patients had gastric cancer and 40 patients die of gastric cancer during the two-year follow-up period.

### 3.4 Toxicity

Forty-six patients received a total of 257 treatment cycles. The median number of cycles administered was 6. The major hematologic toxicities detected included grade I leukocytopenia (23.91%), and grade I thrombocytopenia (23.91%), grade II neutropenia (21.74%). Grade I/II neuropathy was observed in 11 patients (23.9%). Grade I/II mucositis was noted in 4 patients (8.69%). Fifteen patients (32.6%) experienced grade I/II vomiting. Grade I/II neuropathy was observed in 15 patients (32.6%). Eighteen patients (39.13%) experienced grade I/II nausea. The major grade III/IV hematologic toxicities detected included leucocytopenia 6 (13.04%), anemia 6 (13.04%), and neutropenia 7 (15.22%). Only 4 cycle of neutropenic fever was recorded in this study. The more severe nonhematological toxicities observed included grade III diarrhea (2.17%), grade III neurological toxicity (2.17%) and grade III increased creatinine (2.17%). No treatment-related deaths were noted in this study. Toxicities observed during the treatment are provided in Tables 3 and 4.

**Table 3:**
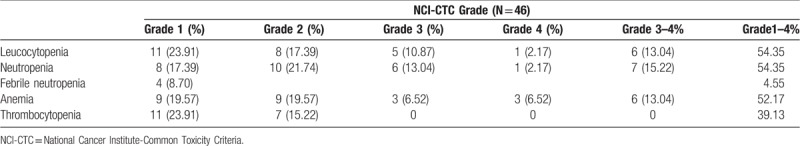
Hematological toxicities.

**Table 4:**
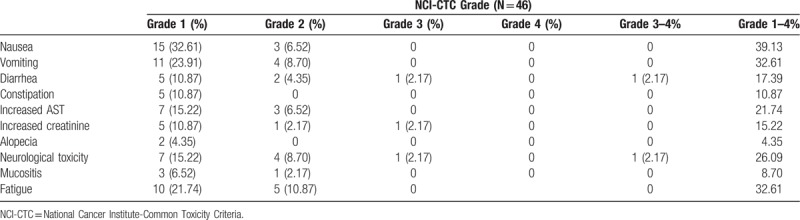
Nonhematological toxicities.

## 4 Discussion

Systemic palliative chemotherapy provides improvement of survival and quality of life for patients with metastatic or advanced gastric cancer. Traditional chemotherapy drugs for gastric cancer including paclitaxel, docetaxel, capecitabine, S-1, cisplatin, and oxaliplatin. Two-drug chemotherapy regimens showed a survival advantage compared to single agent regimens. Three-drug chemotherapy regimens had survival advantages compared to two-drug regimens, but the grade 3 or 4 hematologic toxicity occurs significant higher. Therefore, the two-drug combination regimens of fluorinated pyrimidines and platinum-based drugs were used more than single agent regimens or three-drug regimens associated with a promising response rate and tolerable toxicity. Recently, there have been great advances regarding the carcinogenesis mechanism and treatment.^[15–17]^ But there is still no unified scheme for patients with metastatic or advanced gastric cancer.

The SOX regimen is now being widely adopted for Asian patients with metastatic or advanced gastric cancer. Although some studies of SOX in patients with metastatic or advanced gastric cancer showed tolerable toxicity with 100/130 mg of oxaliplatin, the recommended oxaliplatin dose has not been established yet. In the G-SOX phase III study, oxaliplatin was administered (100 mg/m^2^) on day 1and S-1 was administered (80 mg/m^2^/day) for days 1–14. The response rate of this study was 56% and disease control rate was 84%, PFS was 6.5 months and OS were 16.5 months.^[12]^ The major grade 3/4 toxic effects were neutropenia (22%), thrombocytopenia (13%), anemia (9%), anorexia (6%), and sensory neuropathy (4%). Therefore, activated combination of chemotherapy with less toxic effects is the future direction of development. In our study, the PFS and OS was 4.4 months (95% CI, 3.37–5.36 months) and 10.3 months (95% CI, 8.88–11.3 months) respectively. The 1-year and 2-year survival rate was 41% and 13%, respectively. The ORR and DCR was 30.43% and 76.08%, respectively. In the previous doublet regimen phase III clinical trials, PFS and OS of 8.6 and 4.8 months was reported for SP by the FLAGS trial,^[18]^ 10.5 and 5.6 months was reported for XP regimen,^[19]^ 13.0 and 6.0 months was reported by the SPIRITS trial. Both OS (10.3 months) and TTP (4.4 months) of the biweekly SOX regimen were similar to the results of phase III studies.^[3]^ The PFS of first-line doublet combinations were range from 3.9 to 6.0 months in clinical trials in advanced gastric cancer. The PFS of our study is 4.4 months seems relatively short, but is still in range of the randomized controlled trials. Reasons may explain this phenomenon as follows: Not all of the patients received second-line chemotherapy; only thirty-six patients (78.3%) received second-line chemotherapy. There were ten patients refused to second-line chemotherapy. This study was conducted in real clinical setting that six patients’ ECOG performance statuses were 2. Major of the patients (27/46) were involved in at least two recurrences or distant organ metastasis.

The major grade 3/4 toxic effects were neutropenia (22%), thrombocytopenia (13%), anemia (9%), anorexia (6%), and sensory neuropathy (4%) in G-SOX phase III study.^[12]^ In this study, adverse events of Grade 3/4 included were leukocytopenia (13.04%); anemia (13.04%); neutropenia (15.22%); neurological toxicity (2.17%); and diarrhea (2.17%). The nonhematological toxicities observed included grade 3 diarrhea (2.17%), grade III neurological toxicity (2.17%), and grade III increased creatinine (2.17%). Biweekly SOX in patients with metastatic or advanced gastric cancer using the treatment schedule have shown tolerable toxicity compared to the results of phase III studies. A noteworthy advantage of the biweekly SOX was the reduced hematological toxicities. None patient had grade 3/4 thrombocytopenia, grade 3/4 neutropenia and anemia were less frequent. Noteworthy advantage of less gastrointestinal toxicities was observed, only one patient had grade 3 diarrhea. Less hematological and gastrointestinal toxicities may explicable for more courses of chemotherapy, which lead to 41% patient survival at 1 year and 13% at 2 years. Relatively low proportion (78.3% vs 89%) received the second-line chemotherapy may result in shorter OS compared with G-SOX phase III study (10.3 vs 16.5 months).

Based on our results, the biweekly SOX regimen oxaliplatin in combination with S-1 administered produced encouraging antitumor activity and is associated with acceptable toxicity profile in the metastatic or advanced gastric cancer populations in China. This study is relatively confined to patients in China. The biweekly SOX regimen may represent a useful treatment option and generally for patients who are not tolerating of other intensive chemotherapy regimens. This regimen still needs further evaluation in clinical trials .

## Author Contribution

**Data curation:** Cheng Xiao, Jiong Qian, Yulong Zheng, Fang Song, Qiangfeng Wang, Haiping Jiang, Chenyu Mao.

**Formal analysis:** Cheng Xiao, Yulong Zheng.

**Funding acquisition:** Nong Xu.

**Investigation:** Cheng Xiao, Yulong Zheng, Fang Song.

**Methodology:** Cheng Xiao, Yulong Zheng, Fang Song, Qiangfeng Wang, Haiping Jiang, Chenyu Mao.

**Project administration:** Cheng Xiao, Jiong Qian, Qiangfeng Wang.

**Resources:** Cheng Xiao, Jiong Qian, Qiangfeng Wang, Haiping Jiang, Chenyu Mao.

**Software:** Cheng Xiao, Yulong Zheng, Fang Song.

**Supervision:** Cheng Xiao, Jiong Qian, Qiangfeng Wang.

**Validation:** Cheng Xiao.

**Visualization:** Cheng Xiao.

**Writing – original draft:** Cheng Xiao, Nong Xu.

**Writing – review & editing:** Cheng Xiao, Nong Xu.

Author name: 0000-0001-7499-038

## References

[1] Shen L Shan YS Hu HM Management of gastric cancer in Asia: resource-stratified guidelines. Lancet Oncol 2013;14:e535–47. 24176572 10.1016/S1470-2045(13)70436-4

[2] D’Angelica M Gonen M Brennan MF Patterns of initial recurrence in completely resected gastric adenocarcinoma. Ann Surg 2004;240:808–16. 15492562 10.1097/01.sla.0000143245.28656.15 PMC1356486

[3] Koizumi W Narahara H Hara T S-1 plus cisplatin versus S-1 alone for first-line treatment of advanced gastric cancer (SPIRITS trial): a phase III trial. Lancet Oncol 2008;9:215–21. 18282805 10.1016/S1470-2045(08)70035-4

[4] Bang YJ Van Cutsem E Feyereislova A Trastuzumab in combination with chemotherapy versus chemotherapy alone for treatment of HER2-positive advanced gastric or gastro-oesophageal junction cancer (ToGA): a phase 3, open-label, randomised controlled trial. Lancet 2010;376:687–97. 20728210 10.1016/S0140-6736(10)61121-X

[5] Van Cutsem E Moiseyenko VM Tjulandin S Phase III study of docetaxel and cisplatin plus fluorouracil compared with cisplatin and fluorouracil as first-line therapy for advanced gastric cancer: a report of the V325 Study Group. J Clin Oncol 2006;24:4991–7. 17075117 10.1200/JCO.2006.06.8429

[6] Cunningham D Starling N Rao S Capecitabine and oxaliplatin for advanced esophagogastric cancer. N Engl J Med 2008;358:36–46. 18172173 10.1056/NEJMoa073149

[7] Siegel R Ma J Zou Z Cancer statistics, 2014. CA Cancer J Clin 2014;64:9–29. 24399786 10.3322/caac.21208

[8] Wagner AD Syn NL Moehler M Chemotherapy for advanced gastric cancer. Cochrane Database Syst Rev 2017;8:CD004064. 28850174 10.1002/14651858.CD004064.pub4 PMC6483552

[9] Al-Batran SE Hartmann JT Probst S Phase III trial in metastatic gastroesophageal adenocarcinoma with fluorouracil, leucovorin plus either oxaliplatin or cisplatin: a study of the Arbeitsgemeinschaft Internistische Onkologie. J Clinical Oncol 2008;26:1435–42. 18349393 10.1200/JCO.2007.13.9378

[10] Cunningham D Okines AF Ashley S Capecitabine and oxaliplatin for advanced esophagogastric cancer. N Eng J Med 2010;362:858–9. 10.1056/NEJMc0911925 20200397

[11] Chollet P Schoffski P Weigang-Kohler K Phase II trial with S-1 in chemotherapy-naive patients with gastric cancer. A trial performed by the EORTC Early Clinical Studies Group (ECSG). Eur J Cancer 2003;39:1264–70. 12763215 10.1016/s0959-8049(03)00237-5

[12] Yamada Y Higuchi K Nishikawa K Phase III study comparing oxaliplatin plus S-1 with cisplatin plus S-1 in chemotherapy-naive patients with advanced gastric cancer. Ann Oncol 2015;26:141–8. 25316259 10.1093/annonc/mdu472

[13] Ajani JA Buyse M Lichinitser M Combination of cisplatin/S-1 in the treatment of patients with advanced gastric or gastroesophageal adenocarcinoma: results of noninferiority and safety analyses compared with cisplatin/5-fluorouracil in the First-Line Advanced Gastric Cancer Study. Eur J Cancer 2013;49:3616–24. 23899532 10.1016/j.ejca.2013.07.003

[14] Eisenhauer EA Therasse P Bogaerts J New response evaluation criteria in solid tumours: revised RECIST guideline (version 1.1). Eur J Cancer 2009;45:228–47. 19097774 10.1016/j.ejca.2008.10.026

[15] Kou Y Cheun Y Koag MC Synthesis of 14′,15′-dehydro-ritterazine Y via reductive and oxidative functionalizations of hecogenin acetate. Steroids 2013;78:304–11. 23238516 10.1016/j.steroids.2012.10.021

[16] Kou Y Koag MC Cheun Y Application of hypoiodite-mediated aminyl radical cyclization to synthesis of solasodine acetate. Steroids 2012;77:1069–74. 22583912 10.1016/j.steroids.2012.05.002

[17] Kou Y Koag MC Lee S N7 methylation alters hydrogen-bonding patterns of guanine in duplex DNA. J Am Chem Soc 2015;137:14067–70. 26517568 10.1021/jacs.5b10172 PMC5704973

[18] Ajani JA Rodriguez W Bodoky G Multicenter phase III comparison of cisplatin/S-1 with cisplatin/infusional fluorouracil in advanced gastric or gastroesophageal adenocarcinoma study: the FLAGS trial. J Clin Oncol 2010;28:1547–53. 20159816 10.1200/JCO.2009.25.4706

[19] Kang YK Kang WK Shin DB Capecitabine/cisplatin versus 5-fluorouracil/cisplatin as first-line therapy in patients with advanced gastric cancer: a randomised phase III noninferiority trial. Ann Oncol 2009;20:666–73. 19153121 10.1093/annonc/mdn717

